# Examining the impact of personal freedom on income inequality: Evidence from Sub-Saharan Africa and Western European regions

**DOI:** 10.1371/journal.pone.0302730

**Published:** 2024-04-29

**Authors:** Benard Ohene Kwatia, Godfred Amewu, Christopher Boachie

**Affiliations:** 1 Department of Finance, Accra Institute of Technology, AIT Business School, Kokomlemle, Ghana; 2 Department of Finance, School of Business, University of Ghana, Legon, Ghana; 3 Department of Finance, School of Business, Central University, Miotso, Ghana; University of Education, PAKISTAN

## Abstract

Rising income inequality has recently garnered intensive attention owing to its significance in theory and practice. This has rendered the mechanism through which inequality can be understood, even from a microscopic perspective, to institute proper policies that curtail it important. We contribute to the literature by examining the effect of freedom on income inequality using data from 34 least-free and 18 most-free countries from 2000 to 2020 in Sub-Saharan Africa and Western Europe, respectively. A novel fixed-effects panel quantile regression econometric estimator was employed, and the findings showed a non-linear relationship between personal freedom and income inequality for both regions. Again, both regions reported an inverse relationship between personal freedom and income inequality within the medium-run quantiles. However, a positive relationship was reported in the short-run and long-run quantiles of Sub-Saharan Africa and Western Europe, respectively, whereas the inverse was the same. Finally, the significant adverse relationship was more dominant in Western Europe, implying that high levels of personal freedom in the region explain the low levels of income inequality compared to Sub-Saharan Africa, which has lower levels of personal freedom and higher income inequality. Concerning the findings, it is recommended that policymakers and governments of least- and most-free regions institutionalise personal liberties that support human capital development and establish mechanisms to implement enacted freedoms.

## 1. Introduction

Over the past decades, the global rise in income inequalities has widened within countries [1,2]. To address this challenge, the UN’s 2030 agenda for sustainable development goals (SDG) has focused on ‘leaving no one behind’ [3]; This has directed the discussions on many global policies to confront all forms of inequalities to foster equal access to opportunities, making poverty a primary focus of research in development economics, especially in Africa [4,5]. However, Africa has recorded resilient economic growth among many countries and has enjoyed a recovery since the mid-1990s yet, poverty and human development indicators have not developed as anticipated, and the World Bank report on the attainment of the Millennium Development Goals (MDGs) showed that, poverty has been declining across all regions of the world except Africa [5–8]. Income inequality has empirically been shown to impede growth [9–13]. As asserted by the United Nations Development Programme [14–16], high- and rising-income inequality impedes and halts efforts to eradicate poverty. Thus, had income disparity been lower, African countries would have made considerably greater progress in growth and poverty reduction than they have done over the years [7]. This raises concerns for scholars, governments, and policymakers about understanding the determinants and trends of income disparity within and among countries [17].

Many studies have explored the determinants of income inequalities from several aspects of economic variables, namely; financial development [18], globalization [19], market reforms and private capital [20], technology [21,22], educated labour [23,24], financialization, trade union, labour and welfare [25], remittances and financial development [26], labour institutions [27,28], tourism and governance quality [29], taxes [30] and inflation, export, population, labour force, and unemployment [31]. However, very few empirical studies focus on income inequality and institutions [7,32–35] Besides, these few studies also focus on formal institutional measures that capture political, legal, and economic institutions such as government effectiveness, government spending, rule of law, democracy, regulatory quality, voice and accountability, economic freedom, inflation, external debt, and trade ignoring the informal institutions-income inequality nexus. Given that, informal institutions have been established as a crucial driver of economic relevant behaviour such as financial development [36], social capital and economic transition [37], development [38], bank performance [39] and economic development [40]; it is startling that, empirical studies examining the impact of informal institution on income inequality are still in the minority.

To fill the highlighted existing gap in the literature, this study contributes to the literature as follows: *First*, the study provides further insight into the determinants of income inequality by extending the informal institutions-inequality nexus by proxying informal institutions with personal freedom. The study opts for personal freedom over other informal institutional measures because, personal freedom captures the fundamental human rights of individuals to exercise autonomy, pursue their own goals, express opinions and conduct themselves as one desire, without coercion and undue interference, so far as others are not harmed [41,42]. To this end, the study advances recent studies on informal institutions and income inequality [43,44], which focus on trust and attitudes and informal rule systems, respectively, to determine the relationship between informal institutions and income inequality. This study argues that informal rules, attitudes, and trust are built due to patterns of behaviour based on the internalisation of values, norms and faith in enforcing unwritten rules [45]–primarily influenced by personal freedom. Thus, personal freedom creates an environment of empowerment and autonomy that fosters a sense of confidence and trust [46]. Consequently, the study opines that, without personal freedom, one may not have any trust in social relationships and institutions to spur the needed innovations and competition essential to promoting inclusive policies that address income disparities [47,48].

*Second*, to help draw meaningful conclusions and better understand whether or not Africa’s persistent income inequality flows from personal freedom, the study employs an advanced form of personal freedom variable by Fraser Institute that captures freedom of religion, freedom of relationships, freedom of association, assembly and civil society, freedom of expression and information, and freedom of movement, all within the context of legal protection and security. *Third*, the persistence of income inequality and low levels of freedom in Africa is alarming. In Africa, the average earnings of the top 10% are about 30 times that of the bottom 50%, while in Europe, the difference is about 11 percentage points. This makes Africa record the utmost gap between the average earnings of the top 10% and incomes of the bottom 50% above other regions, even in contrast with high-inequality regions, such as the Middle East, Brazil, and India [49,50]. Again, on personal freedom, Fraser Institute’s 2022 report on the human freedom index identifies sub-Saharan Africa, herein SSA, as the least-free region, while Western Europe, herein West, is identified as the most-free region [51]. For this reason, this study seeks to understand how the dynamics of environments characterised by high and low levels of personal freedom impact income inequality by comparing the two regions to draw empirical conclusions. *Fourth*, as argued by [49,52,53], synthetic income inequality measures such as the Gini coefficient are unable to accurately account for the structure of inequality, especially for heavy-tailed distributions [54] hence, call for the need to go yonder. To help better understand the structure of persistent income inequality in Africa, this study employs the income share of the top 10%, which has been described as key in explaining overall inequality [55], as a proxy for income inequality. *Finally*, the literature on income inequality has mostly examined the relationship with other economic variables by employing a conditional mean model with a fixed effect as seen in studies such as; [22,29,56]; however, this study goes beyond by employing a panel quantile regression estimator [57] to test for non-linearity and understand the heterogeneity in the effect of personal freedom on income inequality at different quantiles.

The remainder of the paper is structured as follows. Section 2 provides a brief review of the literature. Section 3 focuses on the data description and their sources, methodology and analysis approach employed in the study. The empirical results and discussions are presented in Section 4, and Section 5 concludes the study with policy recommendations.

## 2. Literature review

### 2.1 Theoretical review

The initial studies on inequality date back to [58], who associated aggregate demand with income distribution. However, the seminal work of [59] provided an approach to studying income inequality and economic development. The study investigated the relationship between inequality and development. It argued that income inequality tends to increase during the initial stages of economic development while inequality decreases in the progressive stages of development. Nonetheless, as economic development peaks, inequality again increases–a phenomenon commonly described as the Kuznets inverted U-shaped curve and, thereafter, the Kuznets curve. Theoretically, the relationship between personal freedom and inequality emerged years ago. [60] posits that high levels of personal freedom that give individuals the liberty to participate in free trade while upholding their interests will inevitably result in greater equity in the distribution of wealth and opportunities. [61] also argued that expanding people’s capabilities and opportunities–what he termed as the "capabilities approach", can foster narrowing inequality. Thus, equitable distribution of opportunities and resources is enhanced within societies where individuals can pursue their goals and make choices. More recently, [46] distinguished negative freedom–the absence of interference, from positive freedom–the ability to act and attain goals autonomously; and argued that enhancing positive freedom by empowering individuals to overcome barriers that restrict their prospects on economic, societal and political fronts reduces inequality and creates more equitable distribution. As asserted by [62], the humanly devised constraints that structure interactions among groups and individuals, comprising patterns of organization embodied in laws, habits, and customs, and formerly constituted establishments; thus, the rules of the game in society have been described as institutions [63]. Conversely, [63] postulate that a market economy’s efficient and proper functioning depends on suitable institutions. Formal institutions are the explicit set of written and codified laws, contracts, constitutions, official rules, regulations and structures [63–65], while informal institutions emerge gradually and are often deeply entrenched in the beliefs, traditions, culture, trust, norms, and values that shape behaviour and guide social interactions within a community [63,66–68].

### 2.2 Formal institution–income inequality nexus

Prior studies have shown that institutions majorly contribute to rising income inequalities among and within countries [34]. However, the effect documented in the literature has not reached a consensus. [69] investigated how labour market institutions affect inequality, income volatility and labour productivity in Brazil. They found that, in general, Brazil’s social protection system is redistributive. However, it may improve its progressive aspects, notably concerning the pension system, and systemic changes can increase efficiency and reduce inequality. [70] explored the impact of foreign direct investment (FDI) on income inequality in Vietnam using a generalised method of moment (GMM) estimation on a panel of 63 provinces from 2012 to 2018 under the moderating effects of institutions and education. The authors found evidence of non-linearity between FDI and income inequality. Furthermore, FDI increases income inequality in Vietnam, but the effect differs depending on the level of education and institutional quality of the province. [71] used the 2010 China Family Panel Studies (CFPS 2010) to assess income inequality in China. They found that the rise in income inequality in China is driven by structural factors attributable to the Chinese political system. In the studies of [11], income inequality in Jordan was explored using expenditure inequality as a proxy to determine the drivers of income disparity between rural and urban areas and between male-headed households (MHHs) and female-headed households (FHHs). The unconditional quantile regression (UQR) model was employed for household expenditure and income surveys (HEIS, 2017/2018), and the results showed that the education of household heads is a crucial driver of income inequality in Jordan, as well as the geographical location of households. [55] used microdata from the Household Finance and Consumption Survey (HFCS) from 2010 to 2016 to study income and wealth disparities in Malta. The study employed decomposition methods and binary response models and found that income and wealth distribution changed in favour of households in the upper parts of the distributions and persons with tertiary education. Additionally, the decomposition of income inequality revealed labour income as a key driver of income inequality; however, pension income reduces the inequality gap. [72] explored income inequality and institutions in 20th-century America, comparing the current situation to those from the early postwar years. The authors argued that during the early postwar years, government efforts to distribute income were instituted through minimum wages, negotiating frameworks, unions, and progressive taxes. However, in recent years, there has been a reversal in all these institutional patterns. The study concludes that income inequality in the United States can be explained by international trade and skill-based technical change, functioning within a larger institutional narrative. Moreover, the income distribution was found to be strongly influenced by economic institutions.

In their empirical analysis, [73] employed a system-GMM estimator to test the dynamic relationship between institutions, proxied by an extensive array of governance measures, and inequality proxied by the Gini index, to determine if there is reinforcement as predicted by the model. Again, the panel VAR estimator was employed to test the direction of causality between the two through linear feedback analysis on a global sample of 121 countries from 1960 to 2000. The study found that institutions cause income inequality while inequality causes institutions; however, the direction of causality appears to be dominant from inequality to institutions. [7] focused on 52 African countries to understand the dynamics or prevalence of income inequality in the region using macroeconomic and institutional data–the rule of law and transparency. Using panel data models such as ordinary least squares (OLS) and two-stage least squares (TSLS) over the period 1980–2017, they found that, in Africa, income inequality decreases in the least developed and low-income economies while in high-income economies, it increases. Again, the study found that, across convergence clubs, the role of institutions and macroeconomic factors in explaining income inequality is limited and differs. [74] explored a panel of 68 countries comprising 35 developed and 33 developing countries from 2000 to 2019 using the generalised method of moments (GMM) to establish the impact of institutional capacity and fiscal policy on income inequality. The authors found that income tax decreases income inequality in developing countries but not in the total sample. Government size and health and education expenditures were found to be negatively linked to income disparities in developing countries. Institutional capacity measured by corruption and government effectiveness was found not to affect income inequality in the global sample; however, significantly, the coefficients of the interactions between institutional capacity and fiscal policy bear the expected signs, although insignificant. In the work of [75], a first attempt was made to address the question of whether institutions and freedom of the press affect income distribution in BRICS-T (Brazil, Russia, India, China, South Africa, and Turkey) countries from 1993 to 2016, and found that institutional quality and freedom of the press reduce income inequality. An augmented mean group (AMG) was employed to estimate the long-run relationship between institutions, freedom of the press and income inequality. [76] examined the effects of legal, economic, and political institutions on income inequality. Data on 21 OECD advanced countries over two decades were employed in the analysis, and it was found that the impact of society’s utilisation on income inequality is more pronounced than that of unionisation. In the empirical examinations of [77], the authors employed panel data to assess the impact of financial reforms on income inequality in 62 countries from 1973 to 2005. They found that removing subsidised directed credit, improving the securities market policy, decreasing excessively high reserve requirements, and fostering financial reforms foster equal income distributions. They also provide evidence of a non-linear relationship between income inequality and per capita income (Kuznets curve), showing that the size of government is critical to reducing income inequality while trade enhances unequal income distribution.

### 2.3 Informal institution–income inequality nexus

On informal institutions and inequality, [44] compared two highly income-unequal Latin American cities, Santiago in Chile and Medellin in Colombia, to examine how informal institutions impact income distribution. Valenzuela-Levi found that informal rule systems influence income distribution within public transport. [78] examined the link between informality and income inequality using time series and panel data from African countries. The authors estimated a fixed effect model on the panel from 1990 to 2015 and found that informality contributed to decreasing income disparities in Africa. In the empirical examination of [43], the authors employed unbalanced panel data from 1981 to 2014 to examine the impact of trust and attitudes towards income inequality in a global sample. They found that mistrust increases the desire for income inequality in societies where individualism is strongly valued. [79] empirical study was on the relationship between inequality and trust in 22 European countries, covering 216 regions from 2010 to 2014. The study employed multilevel modelling in its analysis and found that differences in income inequality across countries, rather than within countries, account for most of the variation in social trust. [80] investigated the mediating role of the fairness gap (the gap between perceived and preferred inequality) in political trust and income inequality from a cross-country perspective. The author found that a high perceived fairness gap relates to low political trust and moderates the political trust-income inequality nexus. Additionally, when the fairness gap is disaggregated, it becomes clearer that political trust is more closely correlated with changes in inequality perception than with changes in inequality preference. The findings were obtained using pooled ordinary least square regressions and mediation analysis. [81] employed a structural equation model to examine the direction of causality between trust, welfare state and income inequality in 104 countries and found that trust supports welfare state policies that decrease net income inequality. However, the reduced inequality does not increase trust. [56] examine the impact of national culture on income inequality using OLS and 2SLS instrumental variable estimators in a cross-country analysis. The authors found that countries with high levels of collectivist culture tolerate high-income disparity compared with individualistic countries. In support of the above, [82] studied the relationship between culture and income inequality, concentrating on individualistic and collectivistic cultural dimensions. The author found that individualistic countries have significantly lower after-tax income inequality and higher income reallocation than collectivist countries. The study also found that the individualism-redistribution nexus is driven by countries with higher incomes, which seem to be affected by distinct strains of individualism.

## 3. Methodology and data

### 3.1 Data

The study uses data from different sources to investigate a panel of 52 countries comprised of 34 sub-Saharan African countries and 18 Western European countries from 2000 to 2020. The choice of study period and countries employed in the study were dictated by the period for which reliable data were available for the study’s comparative analysis. The regressand in this study is income inequality. As revealed by [49], the pan-African income distribution characterized by extreme levels of inequality with highly high incomes at the top and relatively low incomes at the bottom, calls for the need to go beyond synthetic inequality measures such as the Gini coefficient which is unable to accurately account for the structure of income inequality especially in heavy-tailed distribution as seen in Africa. As such, the study proxy’s income inequality as the pre-tax income share held by the top 10% of the population sourced from the World Inequality Database (WID).

The main regressor is personal freedom, which is sourced from Fraser Institute’s 2022 Human Freedom Index. To help establish whether the causal effect of personal freedom on income inequality is reliable, control variables–trade openness, inflation, general government expenditure, gross domestic product (GDP) growth, and age dependency ratio–deemed significant for the investigation of income inequality are included in the analysis. Trade openness captures the summation of exports and imports normalized by GDP and is expected to reduce inequality [83]. Inflation measures the annual changes in the cost to the average consumer of acquiring a basket of goods and services; at a higher level, it is expected to increase inequality [84]. High levels of inequality call for resource allocation to fund the redistribution of income, thus increasing total government expenditure; as such, inequality is expected to narrow at high levels of government expenditure [85]. The level of economic growth is captured by GDP growth; as growth increases, income inequality is likely to decrease [86]. Age dependency is the sum of the working-age population relative to the sum of the non-working population (those below the age of 15 and above the age of 65). The higher the dependency ratio, the wider the income inequality is expected to widen [87]. All control variables were sourced from World Development Indicators (WDI). Details of variables employed in the study, their coded description, data sources and priori expectation have been described in Table 1 below.

**Table 1:** Variable description and data sources.

Variable	Code	Source	Expected Sign
Income Inequality	INI	World Inequality Database	
Personal Freedom	PEF	Fraser Institute	-
Trade Openness	TOP	World Development Indicators	-
Inflation	INN	World Development Indicators	+
General Government Expenditure	GOV	World Development Indicators	-
Gross Domestic Product	GPG	World Development Indicators	-
Age Dependency	AGD	World Development Indicators	+

### 3.2 Empirical estimation

Most prior studies examining the factors that impact income inequality have often employed a conditional average model with a fixed effect to explore the relationship [7,22,29,80,88].

$${Q(y}_{it}(\tau x_{it},\partial_{i})=+x_{it}^{\prime }\beta (\tau )+\partial_{i},$$ (1)

Where *y*_*it*_ is the logarithm of Income Inequality for country *i* at a year t, $x_{it}^{\prime }\ldots \ldots x_{itp}^{\prime }$ is the px1 vector of the independent variable and ∂_*i*_ is the unobserved country fixed effect, which controls for the time-variant of unobserved heterogeneity. However, this estimation considers only the conditional expectation of the regressand and does not adequately capture the conditional distribution’s overall picture [26]. As the variables employed in this study do not fulfil the assumption of no outliers and normality, we adopt fixed effects panel quantile regression [57] to understand the heterogeneity in the effect of personal freedom on the conditional distribution of income inequality. These heterogeneous effects have been demonstrated to offer valuable information that conventional regression techniques, such as ordinary least squares (OLS), cannot deliver [89]. In contrast to conventional regression, such as OLS, panel quantile regression is more robust in addressing problems of unobserved heterogeneity, heteroscedasticity and outliers in the variables [57,90]. Thus, it uses non-linearity and asymmetry to deal with heterogeneity and endogeneity concurrently [91]. This makes it more appealing for policymakers to do panel analysis, which stratifies the distributive effect of the variables in different quantile distributions [92].

The empirical model is set following Koenker (2004) [57]:

$${Qy}_{it}(\tau x_{it})=\partial_{i}+x_{it}^{\prime }\beta (\tau )+\mu_{ij}j=1,\ldots .,m_{i},\ldots i=1,\ldots n$$ (2)

where *Qy*_*it*_(*τx*_*it*_) represents the *τth* quantile of the dependent variable *x*_*it*_ depicts the vector of explanatory variables, ∂_*i*_ denotes the individual effect, *τ* represents the quantile, *β*(*τ*) is the regression parameter of the *τth* quantile and can be estimated as follows:

$$\underset{\left(\partial ,\beta \right)}{\min}\sum_{k=1}^{q}\sum_{j=1}^{n}\sum_{i=1}^{m_{i}}w_{k}\;\rho_{\tau k}(y_{it}-\partial_{i}-x_{it}^{\prime }\beta (\tau ))$$ (3)

where $\rho_{\tau (u)}=u(\tau -1(u<0)),$ represents the piecewise linear quantile loss function [93]. Weight *w*_*k*_ controls the relative influence of the quantile on the estimation of the ∂_*i*_ which is defined as:

$$\mathrm{If}\;y_{it}-\partial_{i}-x_{it}^{\prime }\beta (\tau )<0,\;\mathrm{then}$$ (4)

$$w_{it}=\tau$$

$$y_{it}-\partial_{i}-x_{it}^{\prime }\beta (\tau )<0,$$ (5)

$$w_{it}=1-\tau$$

From Eqs (4) and (5), we note that quantile regression is a type of weighted regression by setting 1&*τ* positive and negative residuals, respectively [94], which ignores unobserved country heterogeneity. However, owing to the limited yearly observations in our study, we follow [57] estimation approach, in which the unobservable individual effect ∂_*i*_ acts as one of the regression parameters. This is defined as follows;

$$\underset{\left(\partial ,\beta \right)}{\min}\sum_{k=1}^{q}\sum_{j=1}^{n}\sum_{i=1}^{m_{i}}w_{k}\rho_{\tau k}(y_{it}-\gamma_{1\tau }{PER}_{it}{+\gamma_{2\tau }TOP}_{it}+\gamma_{3\tau }{INN}_{it}+{\gamma_{4\tau }GOV}_{it}{+\gamma_{5\tau }GPG}_{it}+\gamma_{6\tau }{AGD}_{it}-\partial_{i})+\lambda \sum_{i=1}^{n}\left|\partial_{i}\right|$$ (6)

Where *λ* is the turning parameter of the individual effect, *w*_*k*_ is the weight the of k^th^ quantile, *m* is the index of the quantile PER, TOP, INN, GOV, GPG and AGD of a nation *i*, time *t* and income inequality *y*.

## 4. Results and discussions

### 4.1 Descriptive statistics

The characteristics of the variables of interest are presented in Tables 2 and 3 for sub-Saharan Africa and Western Europe, respectively. Noticeably, the variance of the variables is relatively large, with TOP, INN and AGD dominating for SSA and TOP, GOV and AGD dominating for West. The summary statistics show that the series are leptokurtic distributions, meaning that the distribution has a fatter tail. This supports [95] argument that normality tests are significantly non-normally distributed.

**Table 2:** Summary statistics for income inequality and personal freedom in sub-Saharan Africa.

Summary statistics	INI	PEF	TOP	INN	GOV	GPG	AGD
Mean	0.520	6.646	65.114	8.374	14.398	4.089	84.459
Median	0.503	6.720	57.497	4.690	13.825	4.470	87.221
Maximum	0.716	8.520	163.619	557.202	43.484	33.629	111.477
Minimum	0.405	3.880	16.352	-16.860	0.952	-36.392	40.421
Std. Dev.	0.067	0.970	28.427	34.995	6.608	4.624	14.449
Skewness	0.872	-0.164	1.003	13.170	1.474	-1.136	-0.989
Kurtosis	3.123	2.312	3.541	186.748	6.295	17.186	4.021
Jarque-Bera	79.931	15.196	112.686	900192.500	510.654	5392.145	129.525
Probability	0.000	0.001	0.000	0.000	0.000	0.000	0.000

Note: INI = Income inequality, PEF = Personal freedom, TOP = Trade openness, INN = Inflation, GOV = General government expenditure, GPG = Gross domestic product growth, and, AGD = Age dependency ratio.

**Table 3:** Summary statistics for income inequality and personal freedom in Western Europe.

**Summary statistics**	**INI**	**PEF**	**TOP**	**INN**	**GOV**	**GPG**	**AGD**
Mean	0.335	9.375	114.457	1.820	20.364	1.756	51.167
Median	0.333	9.400	85.143	1.731	19.985	1.947	51.111
Maximum	0.426	9.690	377.843	12.694	28.148	24.370	62.765
Minimum	0.268	8.140	45.419	-4.478	10.424	-11.325	43.397
Std. Dev.	0.029	0.250	74.456	1.514	3.848	3.327	3.785
Skewness	0.261	-1.113	1.749	1.873	-0.542	0.472	0.422
Kurtosis	2.759	4.979	5.239	15.117	2.930	12.099	3.130
Jarque-Bera	5.217	139.680	271.685	2533.592	18.561	1317.866	11.508
Probability	0.074	0.000	0.000	0.000	0.000	0.000	0.003

Note: INI = Income inequality, PEF = Personal freedom, TOP = Trade openness, INN = Inflation, GOV = General government expenditure, GPG = Gross domestic product growth, and, AGD = Age dependency ratio.

The stationary properties of all variables were checked, and the [96,97], ADF-Fisher Chi-square [98], and PP-Fisher Chi-square [99] results are detailed in Tables 4 and 5 for both regions. Most series are I(1) except for AGD and INN in SSA and AGD, GPG and INN for West that were I(0). Given that cross-sectional dependence is present in our model, the first-generation panel unit root test–which often assumes cross-sectional dependence–may not be adequate to address it [92].

**Table 4:** Unit root test for sub-Saharan Africa.

Variables	INI	PEF	TOP	INN	GOV	GPG	AGD
Levin, Lin & Chu t*	-3.053***	-7.162***	-13.797***	-40.104***	-12.353***	-9.385***	-0.90598
Im, Pesaran and Shin W-stat	-4.215***	-7.669***	-12.812***	-15.804***	-11.608***	-15.947***	0.8297
ADF—Fisher Chi-square	121.873***	184.827***	320.977***	241.154***	256.401***	360.764***	178.553***
PP—Fisher Chi-square	117.985***	305.801***	452.244***	814.149***	523.344***	1721.140***	174.902***

Note: (***), (**), and (*) denote a rejection null hypothesis at 1%, 5%, and 10%, respectively.

**Table 5:** Unit root test for Western Europe.

Variables	INI	PEF	TOP	INN	GOV	GPG	AGD
Levin, Lin & Chu t*	-7.229***	-7.162***	-6.030***	-7.419***	-2.162**	0.539	-6.367***
Im, Pesaran and Shin W-stat	-9.582***	-7.669***	-7.575***	-5.259***	-5.168***	-4.965***	-3.053***
ADF—Fisher Chi-square	156.656***	184.827***	125.502***	91.876***	94.541***	86.758***	81.239***
PP—Fisher Chi-square	316.282***	305.801***	192.466***	101.903***	106.431***	111.020***	8.115

Note: (***), (**), and (*) denote a rejection null hypothesis at 1%, 5%, and 10%, respectively.

In order to account for potential reliance on cross-sectional data, the second-generation panel unit root test was developed [100]. To this end, Breusch-Pagan LM, Pesaran scaled LM, Bias-corrected scaled LM, and Pesaran CD were employed to test for the cross-sectional dependence and the results presented in Tables 6 and 7 for SSA and West, respectively. The results reject the null hypothesis of no cross-sectional dependence at the 1% significance level.

**Table 6:** Test for cross-sectional dependency for sub-Saharan Africa.

Variables	INI	PEF	TOP	INN	GOV	GPG	AGD
Breusch-Pagan LM	4312.090***	2523.873***	2288.063***	1394.467***	2239.438***	1170.076***	6536.727***
Pesaran scaled LM	111.985***	58.600***	51.560***	24.882***	50.108***	18.183***	178.400***
Bias-corrected scaled LM	111.135***	57.750***	50.710***	24.032***	49.258***	17.333***	177.550***
Pesaran CD	3.939***	6.778***	10.998***	19.364***	1.800*	19.810***	32.253***

Note: (***), (**), and (*) denote a rejection null hypothesis at 1%, 5%, and 10%, respectively.

**Table 7:** Test for cross-sectional dependency for Western Europe.

Variables	INI	PEF	TOP	INN	GOV	GPG	AGD
Breusch-Pagan LM	521.644***	1847.133***	1644.550***	1265.014***	1071.966***	1728.785***	1947.559***
Pesaran scaled LM	21.074***	96.847***	85.266***	63.570***	52.534***	90.082***	102.588***
Bias-corrected scaled LM	20.624***	96.397***	84.816***	63.120***	52.084***	89.632***	102.138***
Pesaran CD	4.766***	42.017***	36.905***	32.474***	24.843***	40.367***	29.338***

Note: (***), (**), and (*) denote a rejection null hypothesis at 1%, 5%, and 10%, respectively.

### 4.2 Empirical results

The findings of the relation between personal freedom and income inequality for sub-Saharan Africa (SSA) and Western Europe (West) are presented in Tables 8 and 9, respectively. The results are reported for the quantiles 0.05–0.95 and stratified as; 0.05–0.30, 0.35–0.65 and 0.70–0.95 to represent short-, medium- and long-run effects of the conditional income inequality distribution, respectively. The short-run effect is captured by the immediate impact of a change in personal freedom on income inequality within the lower quantile of 0.05–0.30, while the medium-run effect provides insight into how changes in personal freedom affect income inequality over the moderate period–within the median quantiles of 0.35 to 0.65. The long-run effects capture the impact of personal freedom on income inequality in the long term within the upper quantiles of 0.70 to 0.95.

**Table 8:** Personal freedom and income inequality in sub-Saharan Africa.

Quantiles	PEF	TOP	INN	GOV	GPG	AGD
0.05	1.926**	-3.525***	6.665***	8.560***	1.747*	-2.879***
0.10	4.253***	-3.734***	4.785***	6.892***	0.987	-2.400**
0.15	5.580***	-3.518***	3.730***	6.230***	0.601	-1.342
0.20	0.962	-0.671	1.873*	2.254**	0.626	-0.509
0.25	0.414	0.605	1.768*	3.954***	0.695	0.177
0.30	0.442	-0.619	1.494*	4.610***	0.660	0.094
0.35	-0.484	-0.690	0.874	3.481***	0.311	-0.422
0.40	-0.937*	-0.816	0.746	3.058***	-0.031	-0.253
0.45	-1.774*	-0.999	3.083***	3.865***	0.027	-0.773
0.50	-1.618	-1.186	3.069***	4.884***	-0.116	-0.687
0.55	-1.426	-0.940	2.837***	6.406***	-0.590	-0.858
0.60	-1.444	-1.018	2.767***	7.284***	-0.407	-0.883
0.65	-1.457	-0.696	2.834***	7.735***	-0.220	-1.000
0.70	-2.135**	-1.235	2.714***	8.074***	0.650	-2.067**
0.75	-2.482***	-1.210	2.531***	10.113***	0.820	-2.661***
0.80	-3.327***	-1.458	2.205**	11.571***	0.397	-3.923***
0.85	-1.823*	-2.322**	1.211	10.415***	-0.445	-3.499***
0.90	0.458	-2.264**	0.727	3.126***	-1.519	0.045
0.95	1.476	-2.691***	-0.473	2.177**	-0.939	2.267**

Note: (***), (**), and (*) denote a rejection null hypothesis at 1%, 5%, and 10%, respectively.

**Table 9:** Personal freedom and income inequality in Western Europe.

Quantiles	PEF	TOP	INN	GOV	GPG	AGD
0.05	-2.114**	-0.341	-1.651*	-6.906***	0.724	1.905*
0.10	-1.953**	-0.719	-0.668	-7.066***	0.618	1.734*
0.15	-7.073***	-3.005***	0.197	-8.270***	3.053***	2.953***
0.20	-6.225***	-2.709***	-0.189	-7.541***	2.417**	2.896***
0.25	-4.281***	-2.351**	0.471	-7.358***	1.476*	3.649***
0.30	-3.529***	-1.710*	0.888	-7.107***	0.253	3.954***
0.35	-2.532***	-2.014**	0.441	-6.869***	-0.495	4.190***
0.40	-2.555***	-2.897***	0.476	-7.676***	-1.042	4.006***
0.45	-2.354**	-2.997***	0.735	-7.842***	-1.345	3.701***
0.50	-2.099**	-2.468***	0.511	-7.042***	-1.198	3.828***
0.55	-1.651*	-2.360**	0.895	-6.941***	-1.355	3.660***
0.60	-1.168	-1.763*	0.277	-6.641***	-1.533	3.134***
0.65	-0.517	-1.754*	0.358	-7.720***	-2.328**	3.418***
0.70	-0.064	-1.776*	0.099	-8.273***	-2.187**	2.641***
0.75	1.021	-1.236	0.369	-7.257***	-2.373**	2.045**
0.80	1.285	-1.447	0.243	-7.126***	-2.874***	1.601*
0.85	2.115**	-1.143	0.720	-7.712***	-3.405***	2.292**
0.90	1.820*	-1.677*	0.432	-5.344***	-3.140***	1.271
0.95	1.734*	-3.777***	0.551	-5.728***	-3.070***	-0.288

Note: (***), (**), and (*) denote a rejection null hypothesis at 1%, 5%, and 10%, respectively.

Table 8 shows that the coefficients of personal freedom for SSA are positive across all short-run quantiles, with quantiles Q0.5–0.15 depicting a significant relationship. Meanwhile, as the quantiles progress from Q0.20-Q0.30, the significant positive relationship becomes insignificant. In the medium run, an insignificant negative relationship is reported between personal freedom and income inequality at all quantiles except for Q0.45 and Q0.50, which is significant at the 10% level. As the quantiles move to the long run, the insignificant negative relationship becomes significant within quantiles Q0.70-Q0.85; with Q0.90-Q0.95 also showing an insignificant positive relationship.

The findings have implications that are consistent with the researchers’ expectations. The results confirm the priori expectation of non-linearity in the freedom-inequality nexus in SSA as different depths of the relationship between personal freedom and income inequality are reported at different quantiles. Contrary to expectation, the findings show a significant positive relationship between personal freedom and income inequality in three short-run quantiles (Q0.05-Q0.15). This may imply that in least-free regions like SSA, overly restrictive government policies such as progressive taxation and stringent labour laws may stifle personal freedom but support income redistribution and control inequality. This is in line with [101,102], who found that redistributive policies, such as progressive taxation and strict employment protection legislation, effectively reduce income inequality. Additionally, the study’s findings suggest that lower levels of personal freedom in SSA could trigger the implementation of public policies that contribute to bridging the gap between the rich and poor, such as education, to provide more level ground and access for all, as seen in [103], who argues that without changes in public policy such as education that enhance the human capital development of the relatively disadvantaged in regions characterized by high-income inequality, the trend is likely to persist. Thus, for SSA to narrow income inequality in the short-run, a rigorous public policy targeted at income redistribution should be pursued in the region.

In line with the researchers’ expectations, a negative relationship was reported between personal freedom and income inequality in the medium- to long-run, with 11 out of 13 quantiles (Q0.35-Q0.85) depicting an inverse relationship and four out of six of the long-run quantiles (Q0.70-Q0.85) showing significance. The results highlight the dominance of lower levels of freedom and high levels of inequality in the region and suggest that personal freedom is a crucial driver of income inequality in SSA, especially in the long run. Additionally, the findings imply that the least-free SSA is possibly characterized by policies that foster skewed distribution of services such as healthcare and education and undermine individuals’ ability to freely participate in decision-making processes, which has the potential to enhance innovation, human capital development, and freedom to pursue a career path of interest that could significantly enhance an individual’s earnings ability. This is similar to [104] who argue that the loss of human potential and discriminatory social norms that violate personal freedom are more significant in SSA than in other regions, contributing to the high levels of income inequality in the region. Thus, the findings provide a conclusion to explain that SSA countries will be more equitable in the medium- to long-run when policies that uphold human liberties and foster human capital development are implemented.

The findings from Western Europe are quite divergent from those of sub-Saharan Africa, as shown in Table 8. A negative and significant relationship was recorded between personal freedom and income inequality in the short- and medium-run, with 11 out of 13 quantiles (Q0.05-Q0.55) depicting significance. These findings also align with the researchers’ expectations and suggest that a high level of personal freedom in the West is crucial in determining income inequality. The findings imply that individuals in most-free regions, such as the West, have well-instituted personal freedoms that encourage individuals to take on risk, acquire new qualifications and skills, and pursue innovation and creative entrepreneurship, making them more prone to success with higher chances of increasing their earnings, thereby narrowing the income inequality gap. This confirms [105,106] assertion that high educational and entrepreneurial pursuits, made possible by personal freedom, enhance earning potential to reduce inequality. Additionally, the findings may be confirmed by [107,108], who asserted that societies with high levels of personal freedom, like the West, may be less susceptible to corruption, resulting in more equitable resource distribution and contributing significantly to social mobility by empowering individuals to take advantage of merit-based systems to move up the economic ladder, thereby reducing income inequality. Consequently, the findings suggest that in the short- to medium-run, the West should maintain and improve policies that foster high levels of personal freedom in the region, as it has proven essential for equitable incomes.

Divergently, a positive relationship dominates the long-run quantiles, with three of the six (Q0.85-Q0.95) showing significance. This finding could confirm the gradual deterioration of personal freedom in the West in recent years, as highlighted by [51]. It could result from government interventions such as social welfare programs aimed at transferring wealth from the affluent to the underprivileged in the region [50]. These interventions could be restrictive and impede personal freedom yet foster income equality, confirming [109], who found that more expansive government welfare systems tend to correspond with lower levels of income inequality. Accordingly, in the long-run the West should deepen policies that foster high levels of personal liberties with accompanying opportunities that enhance the earnings of all, especially the underprivileged, to curtail potential inequality widening in the region.

Comparatively, the main conclusions drawn from the above discussions are as follows: Consistent with what the researchers anticipated, the results depicted a non-linear relationship between personal freedom and income inequality for both SSA and the West. Again, both regions reported an inverse relationship between personal freedom and income inequality within the medium-run quantiles. However, on the contrary, a positive relationship was reported in the short-run and long-run quantiles of SSA and West, respectively, while the inverse was also the same. Finally, in contrast, the significant inverse relationship between freedom and inequality was more in the West, implying that the region’s lower levels of income inequality can be explained by its accompanying higher levels of personal freedom. Conversely, the higher levels of income disparity dominant in SSA can be explained by the region’s lower levels of personal freedom. Coherently, the study concludes that, informal institutions proxied by personal freedom is a key determinant of income inequality in both SSA and the West. These findings are consistent with [43,44,56] who also found that, informal institutions are key drivers for income inequality hence, should not be ignored in the debate on the determinants of income disparities.

General comments on the impact of the control variables are also provided. Trade openness reports an adverse relationship with income inequality at all quantiles for both regions. West shows more significance across all quantiles than SSA, which reports significance at the short- and long-run quantiles. This suggests that trade liberalization favours the reduction of income inequality, similar to [83,110], who found that trade openness is associated with a reduction in income inequality, but contrary to [111], who found that trade liberalization contributes to increased wage inequality. In both regions, inflation also reports a positive association with income inequality, with SSA showing more significance in most quantiles. This implies that the high inflation levels in most SSA countries contribute to higher levels of income inequality in the region compared to their counterparts in the West. This finding is similar to [84] findings of a significant positive relationship between inflation and income inequality, suggesting that higher inflation levels worsen inequality. For general government expenditure, an inverse relationship at the 1% significance level was seen in the West, while a positive relationship at the 1% significance level was found in SSA. This implies that government expenditure in the West has an appreciable effect in reducing income inequality as opposed to SSA. Similarly, [16] also document that government expenditure exacerbates inequality in SSA. GDP growth reported a significant negative relationship with inequality in the long-run for the West, but no significant relationship was reported for SSA. The findings highlight how high economic growth within the West environment favours lower inequality, divergent from SSA, which is characterized mainly by lower economic growth and higher inequalities, similar to the findings of [112]. Regarding age dependency, SSA reported an insignificant negative relationship in most quantiles, while West showed a significant positive relationship at all quantiles, with most quantiles reporting significance at the 1% level. This implies that lower levels of age dependency, as seen in the West, support bridging the inequality gap, while higher levels of age dependency due to the ageing population and high fertility rates will worsen income inequality in SSA, similar to the finding of [104].

## 5. Conclusion and policy recommendation

This study examines the relationship between personal freedom and income inequality using data from least- and most-free regions represented by sub-Saharan Africa and Western Europe, respectively. The novel fixed effects panel quantile regression estimation technique was employed on data from 52 countries comprising 34 SSA and 18 West from 2000 to 2020. Additionally, trade openness, inflation, general government expenditure, GDP growth and age dependency were added to the analysis as controls for more reliable causal inferences. First, personal freedom is a crucial determinant of income inequality in both regions; thus, its effect cannot be ignored. In the SSA region, the effect of personal freedom on income inequality moves from a positive relationship to an adverse relationship in the short- to long-run. This implies that while policies aimed at income redistribution should be implemented in the short-run, policies that support personal freedom and encourage human capital development should be strictly adhered to in the medium to long run. Further, we found that in the West, the findings were divergent from those of SSA. The results showed that the impact of personal freedom on income inequality shifted from adverse in the short- to medium-run to a positive association in the long run. This implies that in the short- to medium-run, the West should preserve and enhance policies that support the region’s current level of personal freedom, as they have been shown to be essential for equitable incomes and policies that target promoting high levels of freedom and choice, to maximise the potential of all, especially the poor, be pursued in the long-run.

Our findings have important implications for policymaking. First, SSA policymakers should advocate for constitutionalising personal liberties, such as freedom of expression and information, relationships, association, assembly and civil society, movement, and religion, within the security and legal protection framework. Again, SSA governments and policymakers should put systems in place to ensure that enacted freedoms are practically lived and not just documented. For the West, policymakers should strengthen mechanisms that uphold and maintain high levels of personal freedom, a key driver of equitable income in the region. Public policies aimed at narrowing the inequality gap should not be too stringent to stifle personal liberties; however, they should foster human capital development and personal liberties in both regions.

The current analysis indicates a non-linear relationship between personal freedom and income inequality in SSA and the West. The results proved that the degree of the relationship varied at different quantiles, suggesting a potential threshold impact or a transmission mechanism through which freedom could effectively influence inequality that needs to be further investigated.
